# Author Correction: Engineering programmable material-to-cell pathways via synthetic notch receptors to spatially control differentiation in multicellular constructs

**DOI:** 10.1038/s41467-024-50845-5

**Published:** 2024-08-01

**Authors:** Mher Garibyan, Tyler Hoffman, Thijs Makaske, Stephanie K. Do, Yifan Wu, Brian A. Williams, Alexander R. March, Nathan Cho, Nicolas Pedroncelli, Ricardo Espinosa Lima, Jennifer Soto, Brooke Jackson, Jeffrey W. Santoso, Ali Khademhosseini, Matt Thomson, Song Li, Megan L. McCain, Leonardo Morsut

**Affiliations:** 1 Department of Stem Cell Biology and Regenerative Medicine, Keck School of Medicine of USC, University of Southern California, Los Angeles, CA USA; 2 Eli and Edythe Broad Center, University of Southern California, Los Angeles, CA 90033 USA; 3 Alfred E. Mann Department of Biomedical Engineering, USC Viterbi School of Engineering, University of Southern California, Los Angeles, CA USA; 4 Department of Bioengineering, University of California Los Angeles, Los Angeles, CA USA; 5 Division of Biology and Biological Engineering, California Institute of Technology, Pasadena, CA USA; 6 Broad Stem Cell Center, University of California, Los Angeles, Los Angeles, CA 90095 USA; 7 Jonsson Comprehensive Cancer Center, University of California, Los Angeles, Los Angeles, CA 90095 USA; 8 Present Address: Utrecht University in the lab of Prof. Dr. Lukas Kapitein, Los Angeles, CA 90024 USA; 9 Present Address: Terasaki Institute for Biomedical Innovation (TIBI), Los Angeles, CA 90024 USA

Correction to: *Nature Communications* 10.1038/s41467-024-50126-1, published online 13 July 2024

In this article, the funding line ‘This project has been made possible in part by grant number 2023-332386 from the Chan Zuckerberg Initiative DAF, an advised fund of Silicon Valley Community Foundation’ was omitted. The original article has been corrected.

