# Supplementary material for: Engineering programmable material-to-cell pathways via synthetic notch receptors to spatially control differentiation in multicellular constructs
Source: Nat Commun. 2024 Jul 13;15:5891. doi: 10.1038/s41467-024-50126-1 (PMC11246427; doi:10.1038/s41467-024-50126-1)
Supplement: Supplementary file 1 — Supplemental information [file 41467_2024_50126_MOESM1_ESM.pdf]

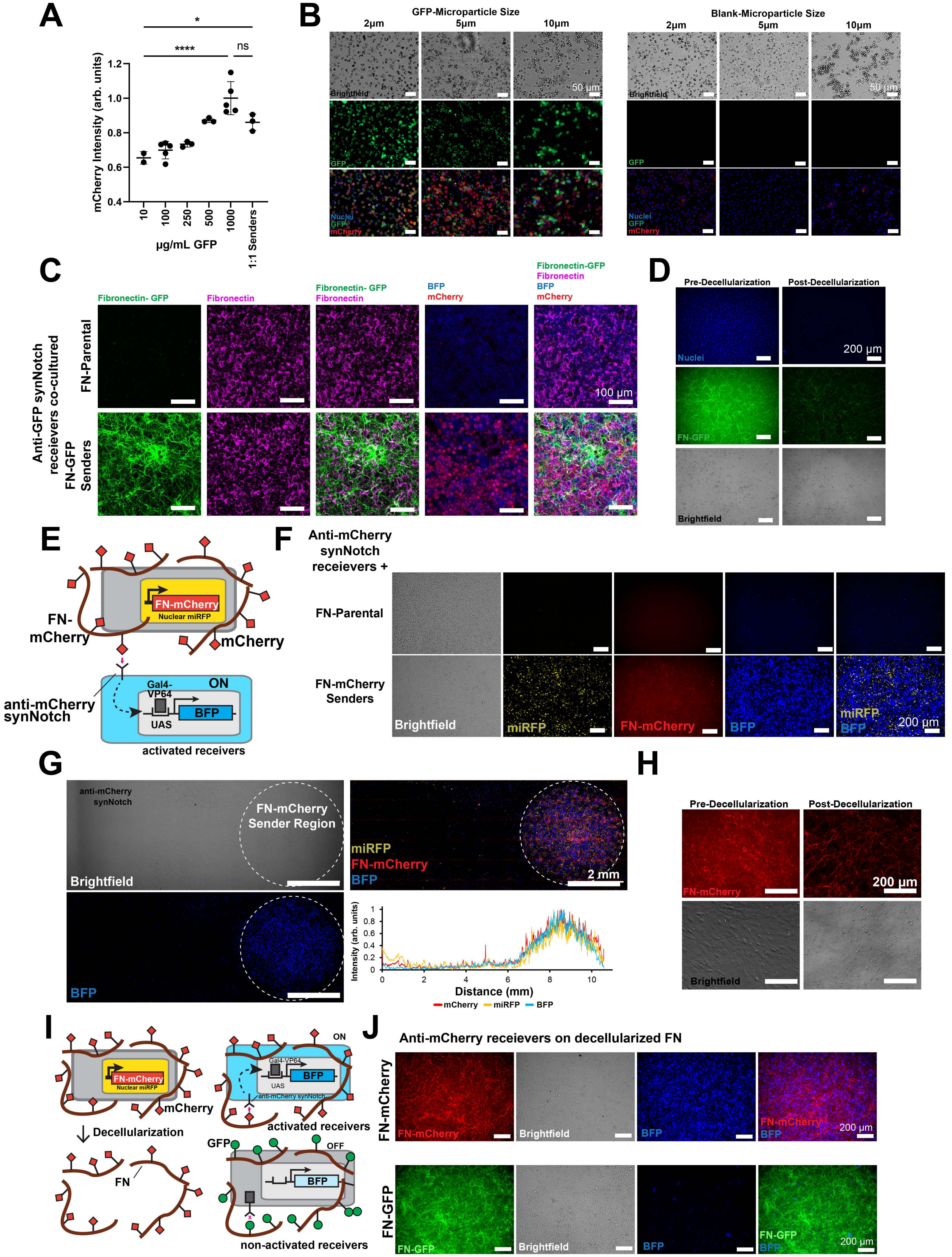
A
B
GFP-Microparticle Size
2μm
5μm
10μm
Blank-Microparticle Size
Brightfield
GFP
Nuclei
mCherry
50 μm
mCherry Intensity (arb. units)
μg/mL GFP
1:1 Senders
C
Fibronectin- GFP
Fibronectin
BFP
mCherry
Anti-GFP synNotch receievers co-cultured
FN-Parental
FN-GFP Senders
100 μm
D
Pre-Decellularization
Post-Decellularization
FN-GFP
200 μm
E
FN-mCherry
Nuclear miRFP
mCherry
anti-mCherry synNotch
Gal4-VP64
ON
UAS
BFP
activated receivers
F
Anti-mCherry synNotch receievers +
FN-Parental
FN-mCherry Senders
miRFP
G
anti-mCherry synNotch
FN-mCherry Sender Region
2 mm
Intensity (arb. units)
Distance (mm)
H
I
Decellularization
FN
GFP
OFF
non-activated receivers
J
Anti-mCherry receievers on decellularized FN
FN-GFP

Figure S1. (A) Average mCherry intensity of activated reporter cells quantified by image analysis following 24-hour co-culture with 5μm GFP microparticles conjugated at the indicated concentrations or GFP sender cells. Data represent mean ± s.d. From left to right, n=2, 5, 3, 3, 5, 3 biological replicates. $p=0.0552$ (ns), $p=0.0191$ (*), $p<0.0001$(****) determined via one-way ANOVA and Tukey's test. (B) Brightfield and fluorescence microscopy images of GFP-conjugated (left) and blank (right) microparticles of varying sizes following 24-hour co-culture with engineered receiver fibroblasts. Scale bars, 50 μm. Experiment was performed two times with similar results. (C) Fluorescence microscopy images of anti-GFP synNotch receiver fibroblasts co-cultured with 3T3 parental fibroblasts (top) or Fibronectin-GFP (FN-GFP) sender fibroblasts for 72 hours, then immunostained for fibronectin (purple). Scale bars, 100 μm. Experiment was performed once. (D) Fluorescence and brightfield microscopy images of FN-GFP sender cells pre- and post-decellularization, showing nuclei (blue) and FN-GFP (green). Scale bars, 200 μm. Experiment was performed four times with similar results. (E) Schematic of sender fibroblasts producing fibronectin-mCherry (FN-mCherry) with a miRFP nuclear tag co-cultured with anti-mCherry/Gal4 synNotch receiver fibroblasts that activate a BFP reporter. (F) Fluorescence microscopy images of anti-mCherry synNotch receiver fibroblasts co-cultured with parental fibroblasts (top) or FN-mCherry sender fibroblasts (bottom) for 48 hours. Scale bars, 200 μm. Experiment was performed four times with similar results. (G) Brightfield and fluorescence microscopy images of anti-mCherry synNotch fibroblasts that activate BFP uniformly seeded on a region of FN-mCherry sender fibroblasts, which were seeded 30 minutes prior as a small droplet. White dotted line indicates the region where FN-mCherry sender fibroblasts were seeded. Line plot represents the normalized fluorescence intensity across the x-axis for one sample. Scale bars, 2 mm. Experiment was performed four times with similar results. (H) Fluorescence and brightfield microscopy images of FN-mCherry sender cells pre- and post-decellularization, showing FN-mCherry (red). Scale bars, 200 μm. Experiment was performed four times with similar results. (I) Schematic of FN-mCherry deposition by FN-mCherry sender cells, decellularization, and reseeding with receiver fibroblasts. Receiver fibroblasts were also reseeded into decellularized FN-GFP deposited by FN-GFP sender cells. (J) Fluorescence microscopy images of anti-mCherry synNotch receiver fibroblasts that activate BFP cultured on decellularized FN-mCherry (top) or FN-GFP (bottom) for 48 hours. Experiment was performed five times with similar results. Source data are provided as a Source Data file.

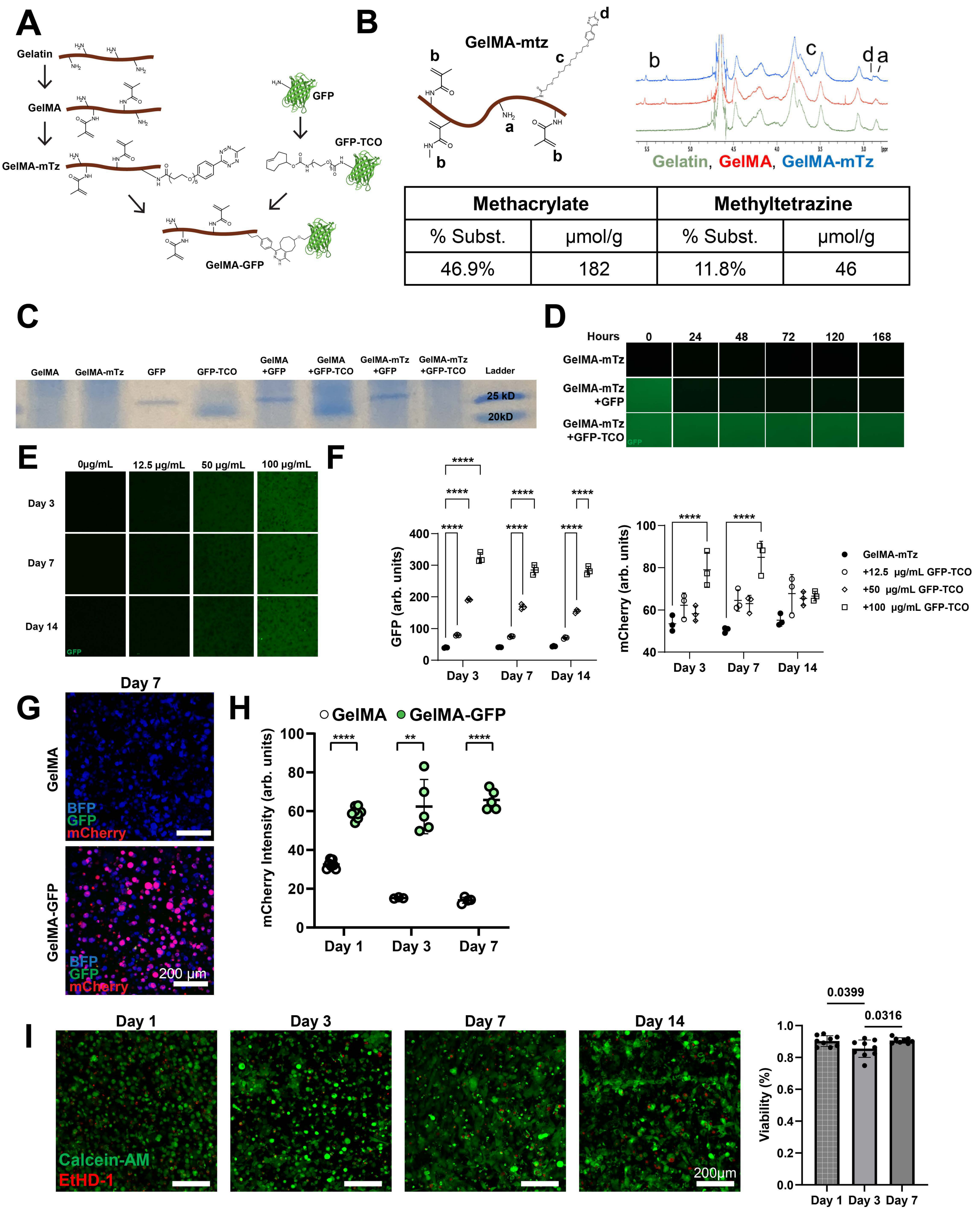

Methacrylate		Methyltetrazine	
% Subst.	μmol/g	% Subst.	μmol/g
46.9%	182	11.8%	46

Figure S2. (A) Schematic of covalent substitutions to gelatin with methacrylate (MA) and methyltetrazine (mTz) to generate GelMA-mTz, and to GFP with trans-cyclooctene (TCO) to generate GFP-TCO. When combined, GelMA-mTz and GFP-TCO generate gelatin methacryloyl (GelMA) conjugated to GFP (GelMA-GFP). (B) Functional groups of GelMA-mTz with corresponding NMR peaks during each step of gelatin modification. Percent substitution and molar content of methacrylate group and methyltetrazine estimated with 1H-NMR. Experiment was performed three times with similar results. (C) Coomassie-blue stained SDS-PAGE gel of indicated combinations of GelMA±mTz with GFP±TCO to determine conjugation. GelMA±mTz is faintly visible due to polydisperse molecular weight. Darker bands indicate GFP (MW: 27 kDa). Protein ladder (right) shown for 25 kDa reference. Experiment was performed four times with similar results. Full gel images are provided in the Source Data file. (D) Fluorescence microscopy images of GFP in GelMA-mTz hydrogels loaded with either no GFP, GFP, or GFP-TCO incubated in PBS at 37°C for indicated hours. Experiments were performed three times with similar results. (E) Fluorescence microscopy images of GFP for anti-GFP synNotch receiver fibroblast-laden GelMA-mTz hydrogels loaded with indicated concentrations of GFP-TCO and cultured for the indicated number of days. (F) Quantification, based on image analysis, of GFP intensity and mCherry intensity for GelMA-mTz hydrogels with indicated concentrations of GFP-TCO and laden with anti-GFP synNotch receiver fibroblasts that activate mCherry, cultured for the indicated number of days. Data represents mean ± s.d, n=3 biological replicates. $p<0.0001$(****) determined via two-way ANOVA and Tukey's test. (G) Z-projection of confocal microscopy images of anti-GFP synNotch fibroblasts that activate mCherry encapsulated in GelMA-mTz hydrogels containing 0 (GelMA) or 50ug/mL GFP-TCO (GelMA-GFP) at Day 7 of culture. Scale bars, 200μm. Related to Fig. 2E. (H) Percent of mCherry expressing receiver cells quantified by image analysis after 1, 3, and 7 days of culture within GelMA or GelMA-GFP hydrogels. From left to right, n=7, 8, 3, 5, 4, 5 biological replicates. $p=0.0013$ (**), $p<0.0001$(****) determined via unpaired two-tailed t-test. (I) Fluorescence images of anti-GFP synNotch fibroblast-laden GelMA hydrogels stained with Live/Dead at the indicated days in culture. Live cells are labeled with Calcein-AM (green) and dead cells are labeled with ethidium homodimer-1 (EtHD-1, red). Scale bars, 200μm. Viability calculated as (number of live cells) / (number of live cells + number of dead cells). Data represents mean ± s.d. From left to right, n=9,9,8 biological replicates. Indicated p-values determined via one-way ANOVA and Tukey's test. Source data are provided as a Source Data file.

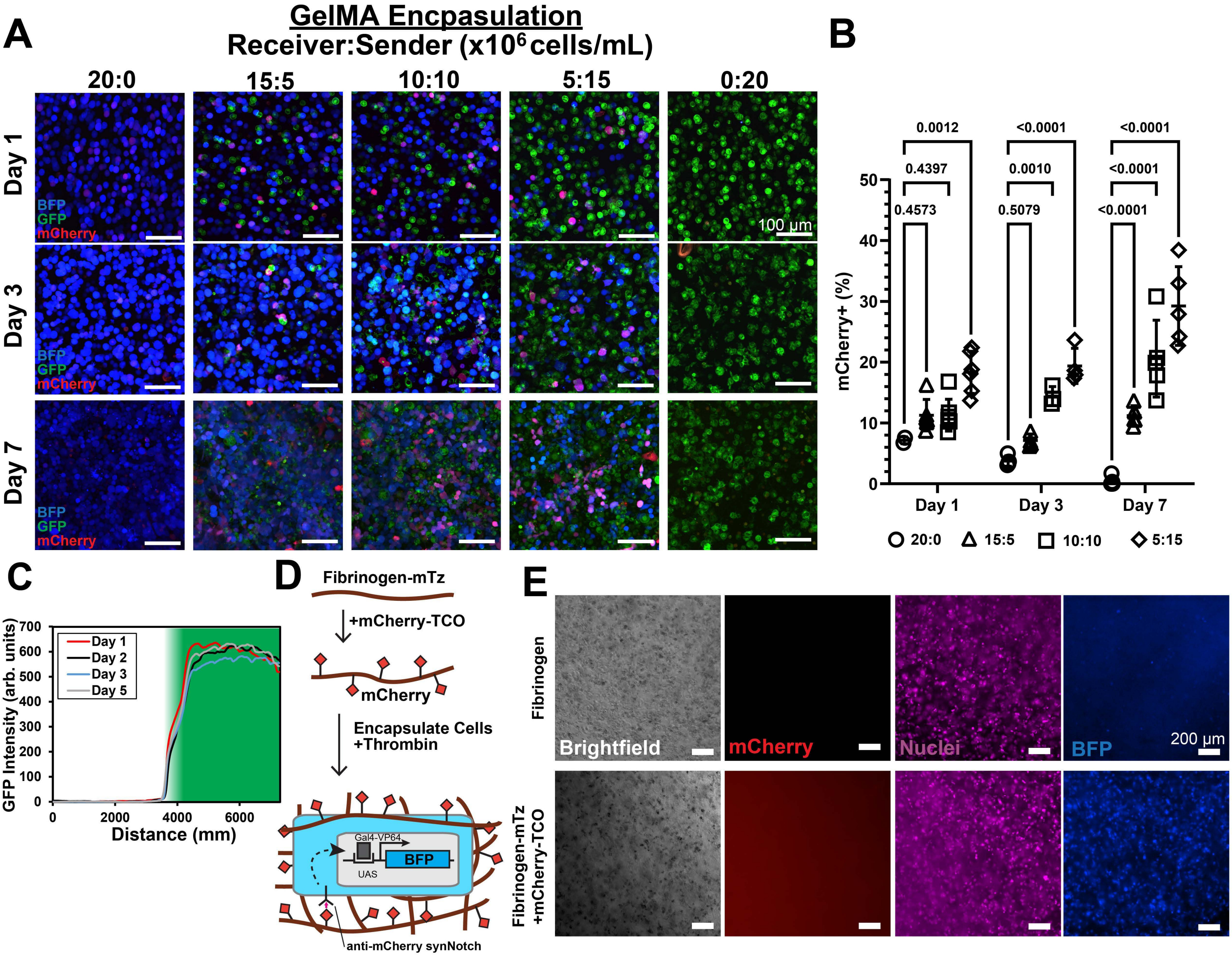
A
GelMA Encpasulation
Receiver:Sender (x10^6 cells/mL)
20:0
15:5
10:10
5:15
0:20
Day 1
Day 3
Day 7
BFP
GFP
mCherry
100 µm
B
mCherry+ (%)
0.0012
0.4397
0.4573
<0.0001
0.0010
0.5079
<0.0001
<0.0001
<0.0001
Day 1
Day 3
Day 7
20:0
15:5
10:10
5:15
C
GFP Intensity (arb. units)
Distance (mm)
Day 1
Day 2
Day 3
Day 5
D
Fibrinogen-mTz
+mCherry-TCO
mCherry
Encapsulate Cells +Thrombin
Gal4-VP64
UAS
BFP
anti-mCherry synNotch
E
Fibrinogen
Fibrinogen-mTz +mCherry-TCO
Brightfield
mCherry
Nuclei
BFP
200 µm

Figure S3. (A) Z-projection of confocal microscopy images of anti-GFP synNotch receiver fibroblasts that activate mCherry co-encapsulated with GFP sender fibroblasts in indicated Receiver:Sender ratios at indicated days following encapsulation. Scale bars, 100 µm. (B) Percent of mCherry expressing receiver cells quantified by image analysis after indicated days of culture with indicated ratios of sender cells. Data represents mean ± s.d. From left to right, n=2,6,7,6,4,4,3,4,5,5,5,5 technical replicates from two biological replicates. Indicated p-values determined via two-way ANOVA and Tukey's test. (C) Plot profile of normalized GFP intensity across the length of a bi-phasic GelMA hydrogel, where only the right portion contains GFP, at indicated days after encapsulation. Green shading indicates the region containing GFP. Data shown are from one replicate, experiment performed two times with similar results. (D) Schematic of Fibrinogen-mTz and mCherry-TCO reaction to generate Fibrinogen-mCherry, which was then used to encapsulate anti-mCherry synNotch fibroblasts that activate BFP. (E) Fluorescence images of anti-mCherry synNotch fibroblasts that activate BFP encapsulated in fibrinogen or fibrinogen-mTz with mCherry-TCO for 24 hours. Nuclei are stained with HSC NuclearMask Deep Red (purple). Scale bars, 200 µm. Experiments were performed four times with similar results. Source data are provided as a Source Data file.

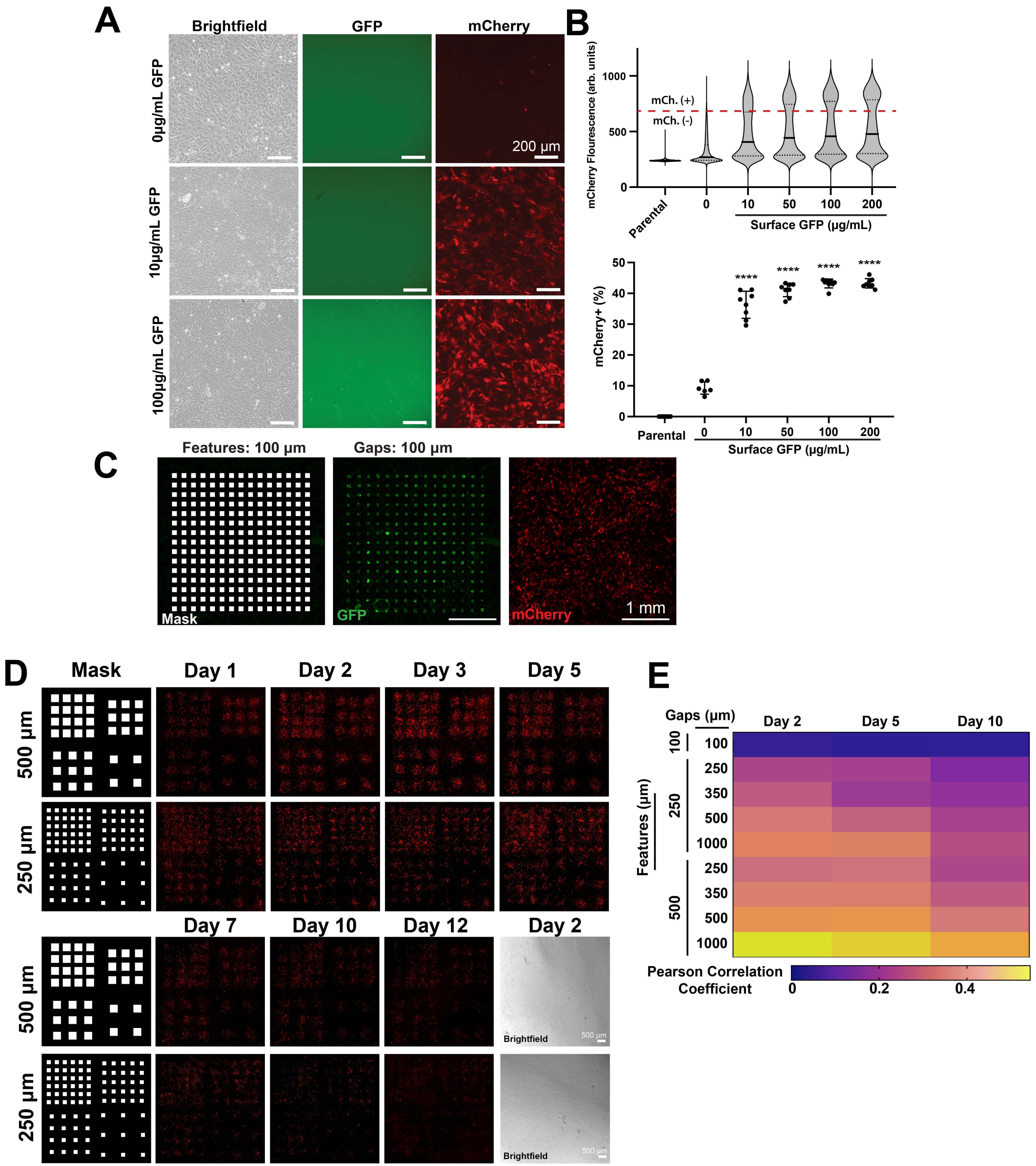
A
Brightfield
GFP
mCherry
0µg/mL GFP
10µg/mL GFP
100µg/mL GFP
200 µm
B
mCherry Flourescence (arb. units)
mCh. (+)
mCh. (-)
Parental
Surface GFP (µg/mL)
mCherry+ (%)
****
C
Features: 100 µm
Gaps: 100 µm
Mask
GFP
mCherry
1 mm
D
Mask
Day 1
Day 2
Day 3
Day 5
Day 7
Day 10
Day 12
500 µm
250 µm
Brightfield
E
Gaps (µm)
Features (µm)
Day 2
Day 5
Day 10
Pearson Correlation Coefficient

Figure S4. (A) Fluorescence microscopy images of anti-GFP synNotch fibroblasts that activate mCherry cultured for two days on PDMS surfaces uniformly microcontact-printed with the indicated concentrations of GFP. Scale bars, 200 µm. (B) Violin plot of mCherry intensity (top) and percent of mCherry expressing cells (bottom), quantified by flow cytometry, for anti-GFP synNotch fibroblasts that activate mCherry cultured for two days on PDMS surfaces microcontact-printed with the indicated concentration of GFP. Parental fibroblasts cultured without GFP were also measured. Dotted line in top panel indicates the threshold value to designate mCherry-positive cells. Top data represent one biological replicate. Bottom data represent mean ± s.d. From left to right, n=7,6,8,8,8,8 biological replicates. $p<0.0001$ (****) compared to synNotch fibroblasts on 0 µg/mL GFP determined via one-way ANOVA and Tukey's test. (C) Fluorescence microscopy images of PDMS surface microcontact-printed with 100 µm GFP squares with 100 µm interspace and seeded with anti-GFP synNotch fibroblasts, cultured for two days. Scale bars, 1mm. (D) Fluorescence and brightfield microscopy images of mCherry for anti-GFP synNotch fibroblasts cultured on 500 µm (top) and 250 µm (bottom) GFP squares with interspacing of 250 µm (top left), 350 µm (top right), 500 µm (bottom left), and 1000 µm (bottom right) for indicated days. Scale bars, 500µm. (E) Heatmap of Pearson correlation coefficient between mCherry and pattern mask of each square width and interspace length at Days 2, 5, and 10. Color map represents the mean coefficient. n=7 for 500x1000µm day 10, 500x350µm days 2, 5, and 10, 500x250µm days 2, 5, and 10, 250x1000µm days 2 and 5, 250x350µm day 2. N=8 for 500x1000µm days 2 and 5, 500x500µm days 2, 5, and 10, 250x1000µm day 10, 250x500µm days 2, 5, and 10, 250x350µm days 5 and 10, 250x250µm days 2, 5, and 10, 100x100µm days 2, 5, and 10. All conditions except 100x100µm pattern scored $p<0.0001$ determined via 2way ANOVA and Dunnett's multiple comparisons test compared to scrambled negative control. Source data are provided as a Source Data file.

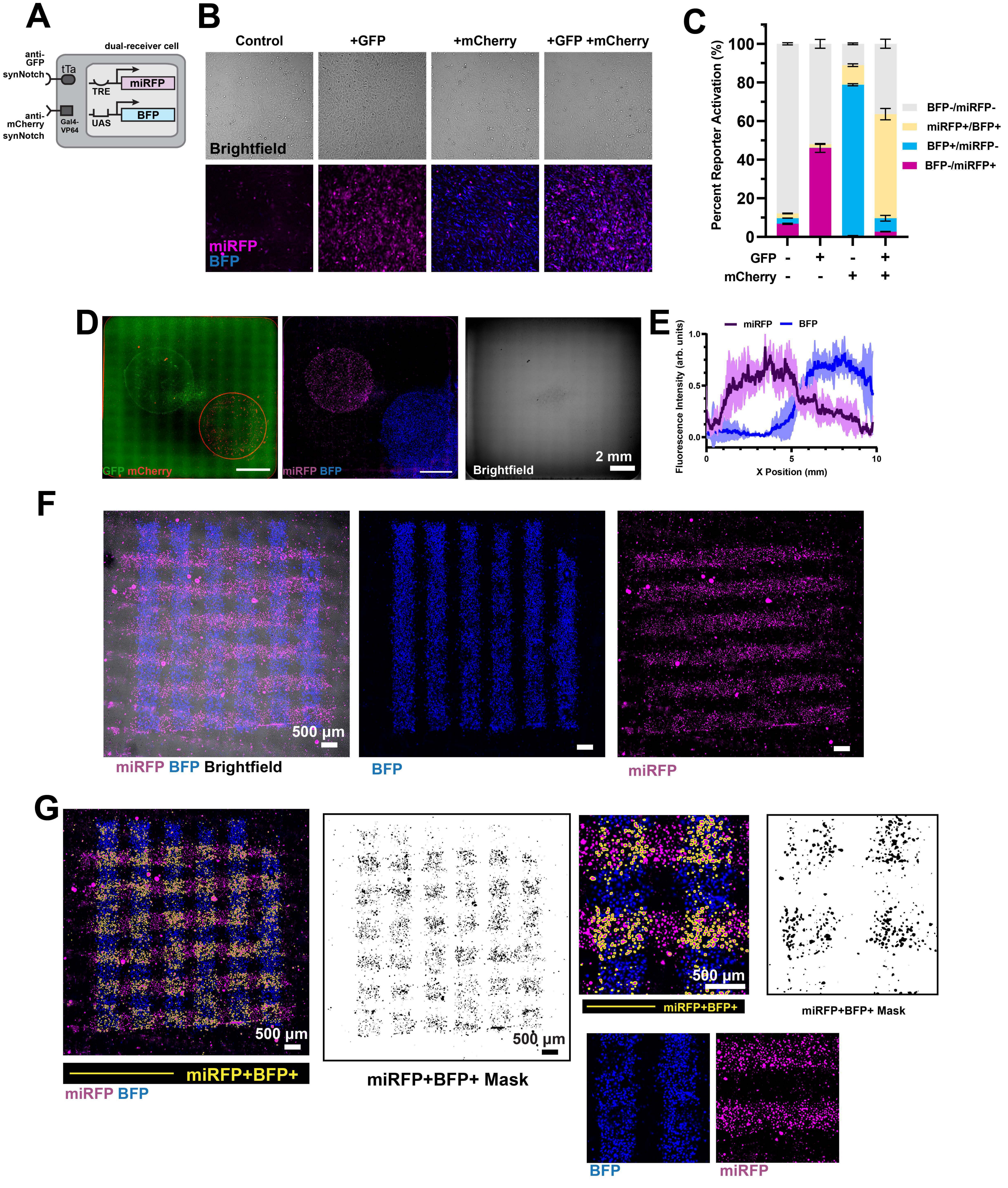

A
dual-receiver cell
anti-GFP synNotch
tTa
TRE
miRFP
anti-mCherry synNotch
Gal4-VP64
UAS
BFP
B
Control
+GFP
+mCherry
+GFP +mCherry
Brightfield
miRFP
BFP
C
Percent Reporter Activation (%)
100
80
60
40
20
0
BFP-/miRFP-
miRFP+/BFP+
BFP+/miRFP-
BFP-/miRFP+
GFP - + - +
mCherry - - + +
D
GFP mCherry
miRFP BFP
Brightfield
2 mm
E
miRFP
BFP
Fluorescence Intensity (arb. units)
1.0
0.5
0.0
0
5
10
X Position (mm)
F
500 µm
miRFP BFP Brightfield
BFP
miRFP
G
500 µm
miRFP+BFP+
miRFP BFP
500 µm
miRFP+BFP+ Mask
500 µm
miRFP+BFP+
miRFP+BFP+ Mask
BFP
miRFP

Figure S5. (A) Schematic of dual-reporter receiver fibroblast (L929) with anti-GFP synNotch that activates miRFP reporter gene and orthogonal anti-mCherry synNotch that activates BFP. (B) Brightfield and fluorescence microscopy images of dual-reporter receiver fibroblasts cultured on plates adsorbed with no ligand (control), GFP, mCherry, or both GFP and mCherry for one day. Scale bars, 200 µm. (C) Percent reporter activation of dual-reporter receiver fibroblasts measured via flow cytometry on culture surfaces that were uniformly adsorbed with the indicated ligands. Data represent mean ± s.d, n=4 biological replicates. (D) Fluorescence microscopy images of GFP and mCherry droplets adsorbed on a culture plate and subsequent expression of miRFP and BFP by dual-receiver reporter fibroblasts cultured on the plate for two days. Brightfield image shows uniform cell coverage. Scale bars, 2mm. (E) Normalized profile plots of miRFP and BFP intensity across the x-axis for images shown in (D). Solid line represents the mean, shading represents s.d. n=4 biological replicates. (F) Fluorescence and brightfield microscopy images of miRFP and BFP by dual-reporter receiver fibroblasts cultured for two days on perpendicular GFP and mCherry rows. Scale bars, 500 µm. (G) Dual-positive miRFP and BFP masks, created using ImageJ image calculator AND function for BFP and miRFP signal above a threshold. Mask was superimposed onto fluorescence images and highlighted with a yellow border to indicate BFP+/miRFP+ cells. Scale bars, 500 µm. Source data are provided as a Source Data file.

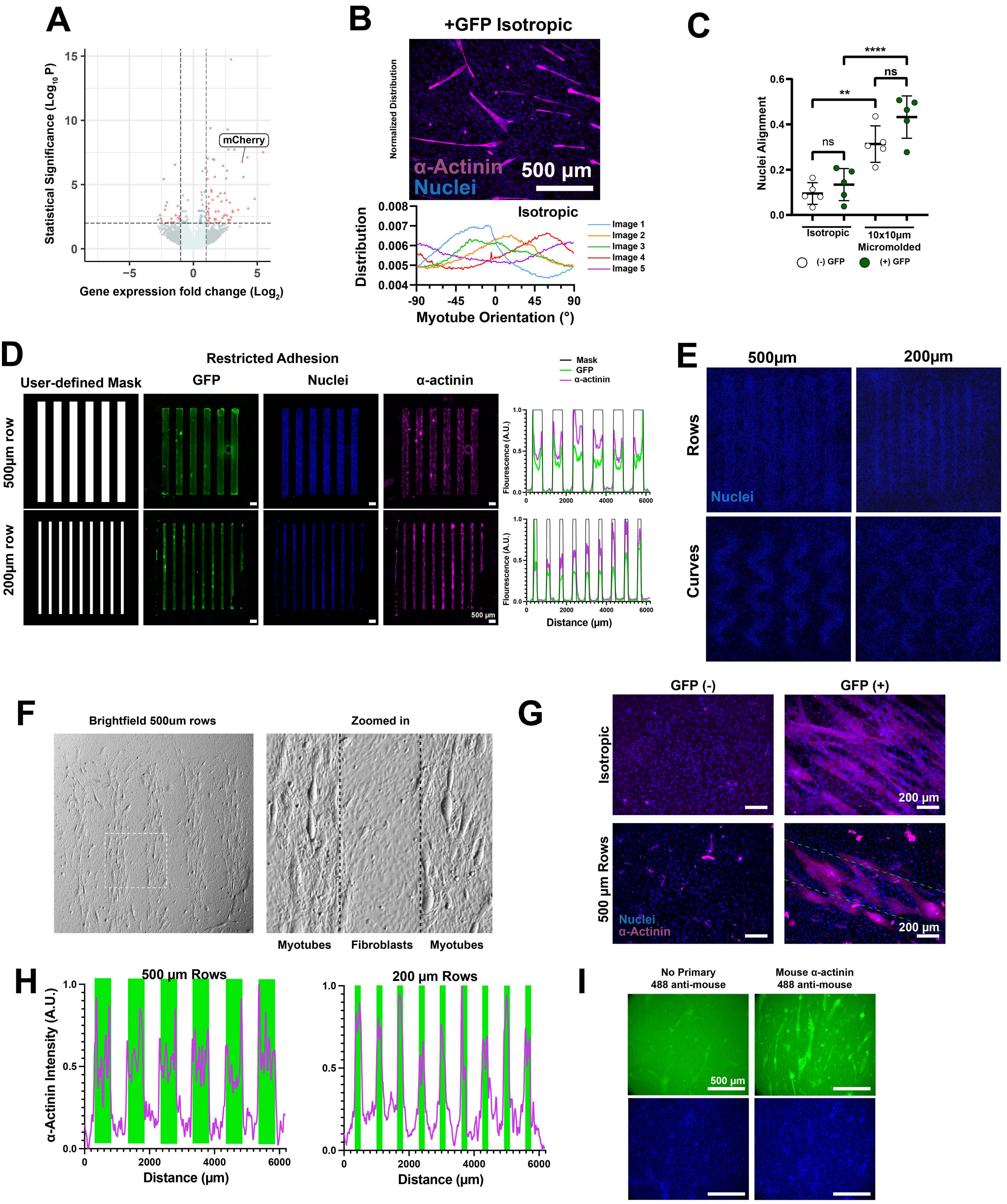
A
Statistical Significance (Log10 P)
Gene expression fold change (Log2)
mCherry
B
+GFP Isotropic
Normalized Distribution
α-Actinin
Nuclei
500 μm
Isotropic
Distribution
Myotube Orientation (°)
Image 1
Image 2
Image 3
Image 4
Image 5
C
Nuclei Alignment
ns
**
****
Isotropic
10x10μm Micromolded
(-) GFP
(+) GFP
D
Restricted Adhesion
User-defined Mask
GFP
Nuclei
α-actinin
500μm row
200μm row
Mask
Flourescence (A.U.)
Distance (μm)
500 μm
E
500μm
200μm
Rows
Curves
Nuclei
F
Brightfield 500um rows
Zoomed in
Myotubes
Fibroblasts
Myotubes
G
GFP (-)
GFP (+)
Isotropic
500 μm Rows
Nuclei
α-Actinin
200 μm
H
500 μm Rows
200 μm Rows
α-Actinin Intensity (A.U.)
Distance (μm)
I
No Primary
488 anti-mouse
Mouse α-actinin
488 anti-mouse
500 μm

Figure S6. (A) Volcano plot of gene expression, quantified by bulk RNA sequencing, showing differentially expressed genes in anti-GFP synNotch fibroblasts that activate a mCherry reporter on GFP compared to off GFP. n=2 biological replicates. (B) (top) Fluorescence microscopy image of anti-GFP synNotch fibroblasts that activate myoD cultured for seven days on isotropic gelatin hydrogel conjugated with 100 µg/mL GFP and then immunostained for α-actinin (purple). Scale bar, 500µm. (bottom) Orientation of α-actinin stained myotubes on isotropic gelatin hydrogel conjugated with 100 µg/mL GFP, quantified by image analysis. Each line represents one image from a single tissue. Experiment performed five times with similar results. (C) Quantification of nuclei alignment for anti-GFP synNotch fibroblasts that activate myoD on the indicated hydrogels. Data represent mean ± s.d, n=5 biological replicates. ns, p=0.8435 for isotropic tissues; ns, p=0.0972 for micromolded tissues; p=0.0015(**), p<0.0001(****) determined via one-way ANOVA and Tukey's test. (D) User-defined patterns and resulting fluorescence microscopy images of microcontact-printed mixture of fibronectin and GFP. Anti-GFP myoD receiver fibroblasts were cultured on surfaces for three days and then stained for sarcomeric α-actinin (purple) and DAPI (blue). Scale bars, 500 µm. Profile plot of normalized GFP intensity and α-actinin immunosignal intensity on corresponding patterns. (E) Fluorescence microscopy images of nuclei stained in anti-GFP myoD receiver fibroblasts cultured for three days on surfaces uniformly coated with fibronectin, then microcontact-printed with the indicated GFP pattern. Images are from the same tissues shown in Fig. 5I. (F) Brightfield microscopy image of anti-GFP myoD receiver fibroblasts cultured on 500 µm GFP rows for three days. Region indicated by dotted white line on the left image is shown at higher magnification in the right image. Cells with a myotube morphology are visible on patterned GFP. (G) Fluorescence microscopy images of anti-GFP myoD receiver fibroblasts cultured on the indicated substrates. α-actinin stained cells on isotropic or 500µm row GFP patterns. Scale bars, 200µm. (H) Profile plots of normalized α-actinin immunosignal intensity on corresponding patterns. Green lines indicate regions patterned with GFP. (I) Fluorescence microscopy images of anti-GFP myoD receiver fibroblasts cultured on GFP for three days and immunostained with anti-mouse Alexa Fluor 488 secondary antibody only (left) or mouse α-actinin antibody and anti-mouse Alexa Fluor 488 secondary antibody (right). Scale bar is 500µm. Source data are provided as a Source Data file.

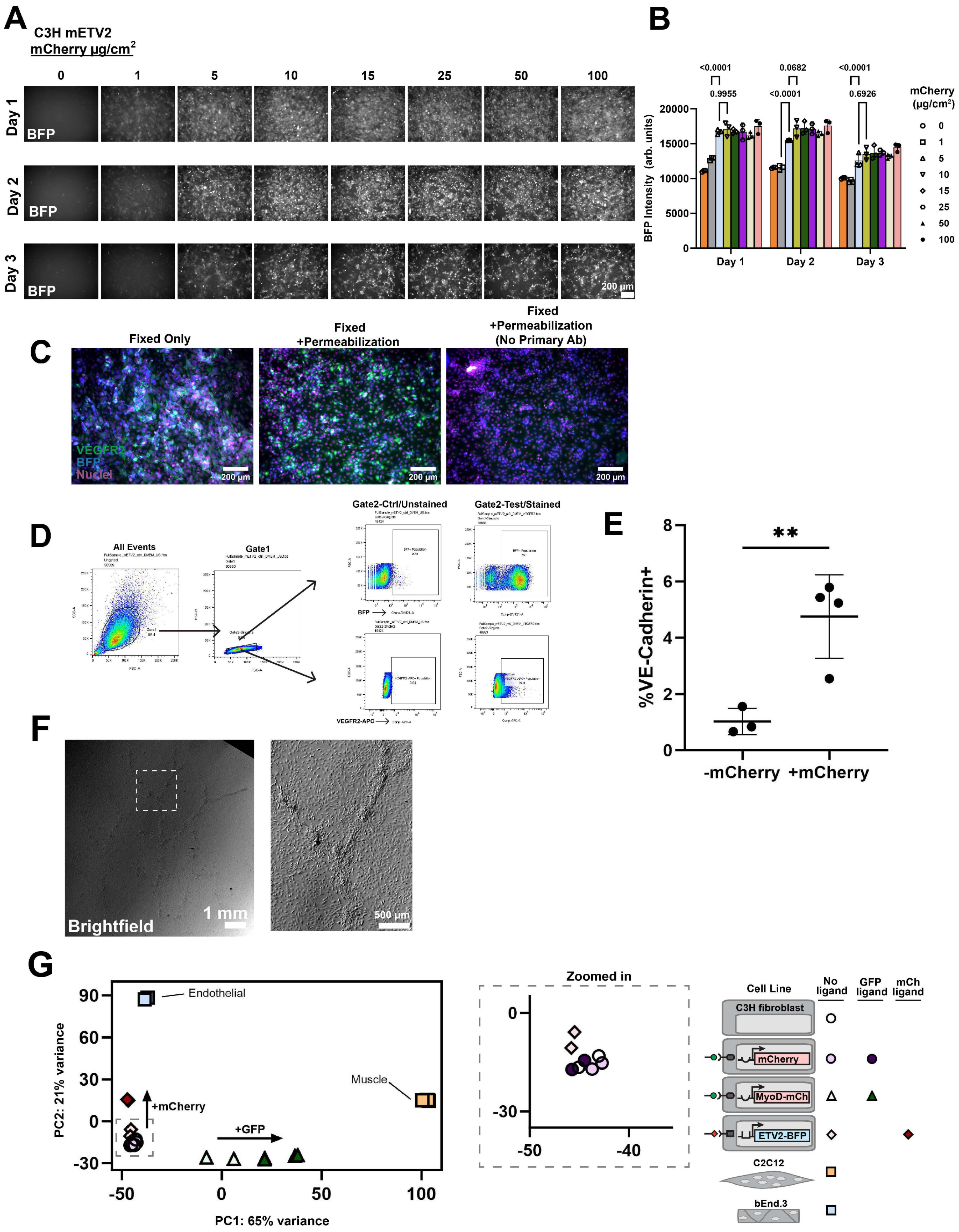
A
C3H mETV2
mCherry µg/cm2
0
1
5
10
15
25
50
100
Day 1
Day 2
Day 3
BFP
200 µm
B
<0.0001
0.9955
0.0682
<0.0001
<0.0001
0.6926
mCherry (µg/cm2)
BFP Intensity (arb. units)
Day 1
Day 2
Day 3
C
Fixed Only
Fixed +Permeabilization
Fixed +Permeabilization (No Primary Ab)
VEGFR2
BFP
Nuclei
200 µm
D
All Events
Gate1
Gate2-Ctrl/Unstained
Gate2-Test/Stained
BFP
VEGFR2-APC
E
%VE-Cadherin+
-mCherry
+mCherry
**
F
Brightfield
1 mm
500 µm
G
Endothelial
Muscle
+mCherry
+GFP
PC2: 21% variance
PC1: 65% variance
Zoomed in
Cell Line
No ligand
GFP ligand
mCh ligand
C3H fibroblast
mCherry
MyoD-mCh
ETV2-BFP
C2C12
bEnd.3

Figure S7. (A) Fluorescence microscopy images of anti-mCherry synNotch fibroblasts that activate ETV2 and BFP cultured for the indicated number of days on plates absorbed with indicated concentrations of mCherry. BFP signal shown in grayscale. Scale bar, 200μm. (B) Quantification of normalized BFP intensity for anti-mCherry synNotch fibroblasts that activate ETV2 and BFP cultured for the indicated number of days on plates absorbed with indicated concentrations of mCherry. Data represent mean ± s.d, n=3 biological replicates. Indicated p-values determined via two-way ANOVA and Tukey's test. (C) Fluorescence microscopy images of anti-mCherry synNotch ETV2-BFP fibroblasts seeded on plate adsorbed with 15 μg/cm2 mCherry and exposed to indicated conditions to validate VEGFR2 antibody for immunostaining. Left: cells were fixed and incubated with VEGFR2 primary antibody and corresponding secondary antibody, but not permeabilized. Center: cells were fixed, permeabilized, and incubated with VEGFR2 primary antibody and corresponding secondary antibody. Right: cells were fixed, permeabilized, and incubated with secondary antibody only (no VEGFR2 antibody). Inducible BFP reporter shown in blue and nuclei stained with HSC NuclearMask Deep Red. Scale bars, 200μm. (D) Flow cytometry gating strategy to exclude debris (Gate 1), isolate singlets (Gate 2), and quantify BFP-expressing cells (top) or VEGFR2 expressing cells (bottom). (E) Measurement of CDH5 (VE-Cadherin) via immunostaining and flow cytometry for anti-mCherry synNotch ETV2-BFP fibroblasts cultured on plates without or with mCherry. Data represent mean ± s.d, n=3 biological replicates for -mCherry and n=4 biological replicates for +mcherry. p=0.0093(**) determined via unpaired two-tailed t-test. (F) Day 3 brightfield image of spatial endothelial-progenitor cell activation on vascular-like pattern, corresponding to the fluorescent microscope images shown in main Fig. 6H. Dotted white line indicates the region of interest enhanced in the following panel. Scale bars, 1mm and 500μm, respectively. (G) Principal Component Analysis (PCA) comparing the transcriptome, determined by bulk RNA sequencing, of unmodified C3H fibroblasts, cell type-specific positive control cell lines (C2C12 and Bend.3), and receiver cells that express mCherry; MyoD and mCherry; or ETV2 and BFP in the presence or absence of their corresponding ligand. n=2 biological replicates for all conditions except +GFP myoD receiver cells and C2C12, which were both n=4 biological replicates. Source data are provided as a Source Data file.

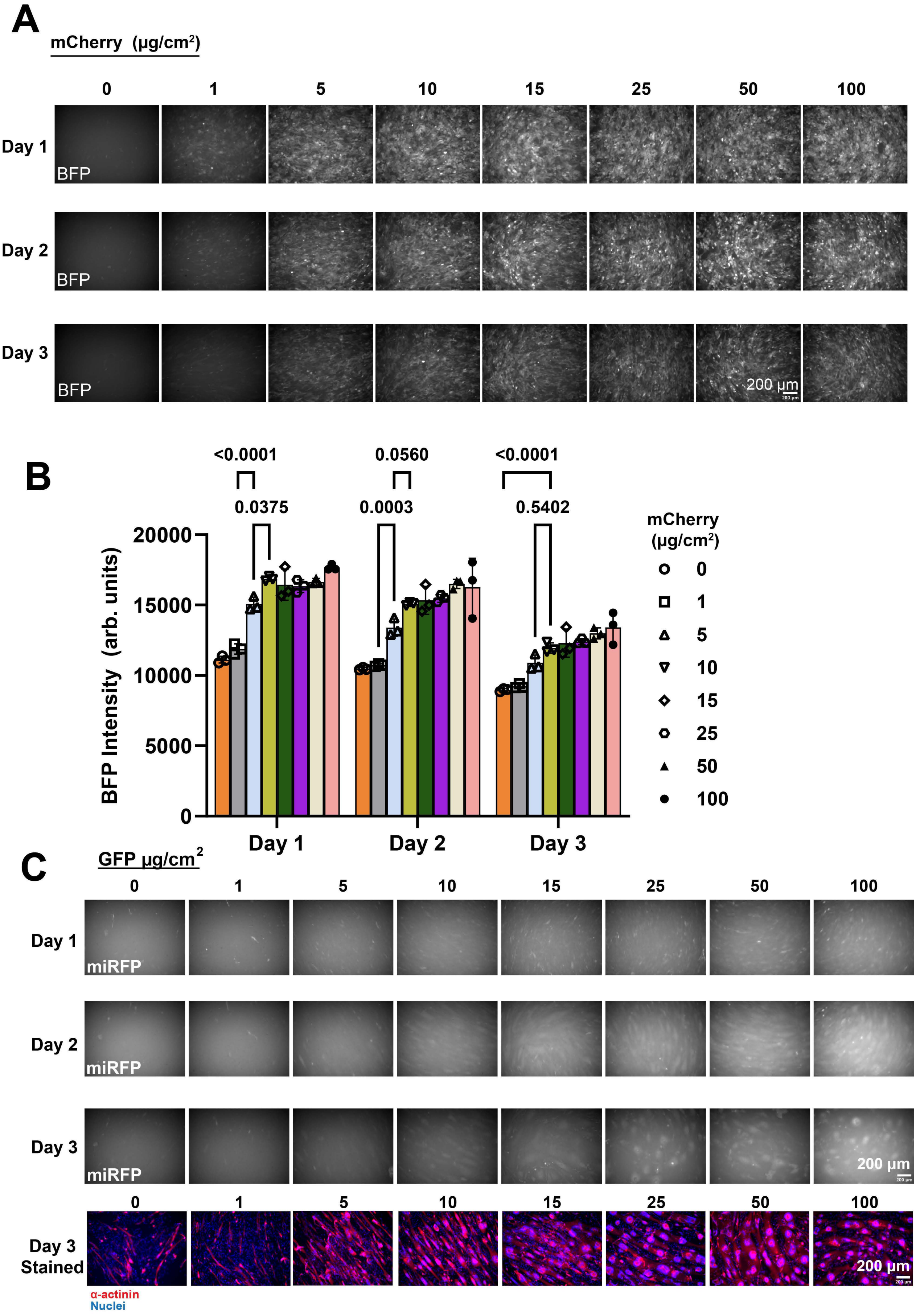
A
mCherry (µg/cm2)
0
1
5
10
15
25
50
100
Day 1
Day 2
Day 3
BFP
200 µm
B
<0.0001
0.0375
0.0560
0.0003
<0.0001
0.5402
BFP Intensity (arb. units)
20000
15000
10000
5000
0
Day 1
Day 2
Day 3
mCherry (µg/cm2)
0
1
5
10
15
25
50
100
C
GFP µg/cm2
0
1
5
10
15
25
50
100
Day 1
Day 2
Day 3
miRFP
200 µm
Day 3 Stained
α-actinin
Nuclei

Figure S8. (A) Fluorescence microscopy images of anti-GFP and anti-mCherry synNotch dual-lineage fibroblasts with orthogonally inducible MyoD-miRFP and ETV2-BFP, respectively, cultured for the indicated number of days on plates adsorbed with indicated concentrations of mCherry. BFP signal shown in grayscale. Scale bar, 200μm. (B) Quantification of normalized BFP intensity for anti-GFP and anti-mCherry synNotch dual-lineage fibroblasts with orthogonally inducible MyoD-miRFP and ETV2-BFP, respectively, cultured for the indicated number of days on plates adsorbed with indicated concentrations of mCherry. Data represent mean ± s.d, n=3 biological replicates. Indicated p-values determined via two-way ANOVA and Tukey's test. (C) Fluorescence microscopy images of anti-GFP and anti-mCherry synNotch dual-lineage fibroblasts with orthogonally inducible MyoD-miRFP and ETV2-BFP, respectively, cultured for the indicated number of days on plates adsorbed with indicated concentrations of GFP. miRFP signal shown in grayscale. Scale bar, 200μm. Experiment was performed four times with similar results. Bottom row, corresponding tissues were fixed and immunostained for α-actinin (red) after three days of culture. Source data are provided as a Source Data file.

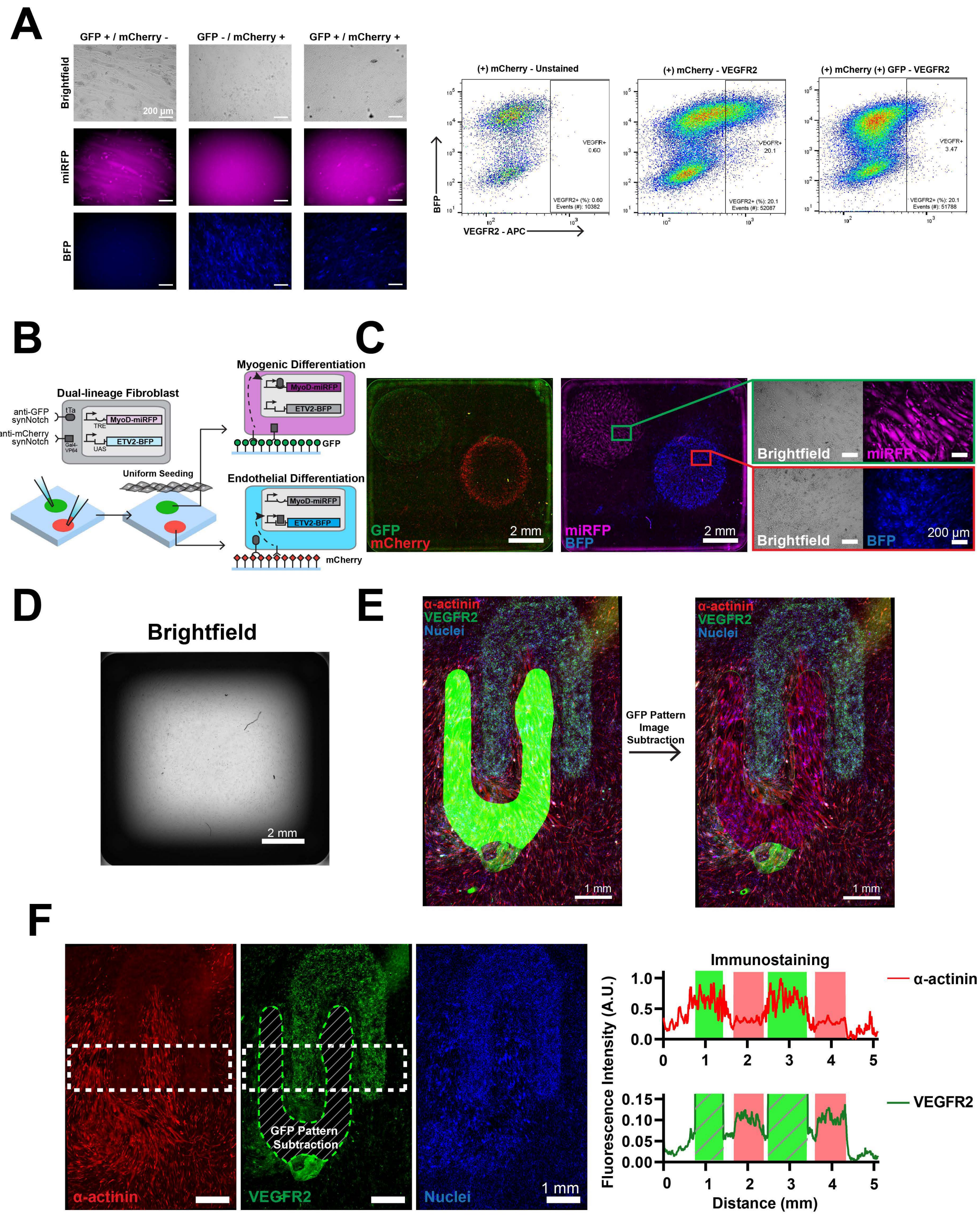

A
GFP + / mCherry -
GFP - / mCherry +
GFP + / mCherry +
Brightfield
miRFP
BFP
200 µm
(+) mCherry - Unstained
(+) mCherry - VEGFR2
(+) mCherry (+) GFP - VEGFR2
BFP
VEGFR2 - APC
B
Dual-lineage Fibroblast
anti-GFP synNotch
anti-mCherry synNotch
MyoD-miRFP
ETV2-BFP
Uniform Seeding
Myogenic Differentiation
Endothelial Differentiation
GFP
mCherry
C
GFP
mCherry
2 mm
miRFP
BFP
Brightfield
200 µm
D
Brightfield
2 mm
E
α-actinin
VEGFR2
Nuclei
GFP Pattern Image Subtraction
1 mm
F
GFP Pattern Subtraction
Nuclei
1 mm
Immunostaining
α-actinin
VEGFR2
Fluorescence Intensity (A.U.)
Distance (mm)

Figure S9. (A) Left: Brightfield and fluorescence microscopy images of anti-GFP and anti-mCherry synNotch dual-lineage fibroblasts with orthogonally inducible MyoD-miRFP and ETV2-BFP, respectively, cultured on plates uniformly adsorbed with GFP only, mCherry only, or both GFP and mCherry. Right: Flow cytometry panel (x-Axis: VEGFR2-APC, y-Axis: BFP) of dual-lineage fibroblasts seeded on plate adsorbed with 15 µg/cm2 mCherry and then unstained (left) or stained for VEGFR2 (center). Dual-lineage fibroblasts seeded on plate adsorbed with 15 µg/cm2 of both GFP and mCherry and then stained for VEGFR2 (right). Experiment was performed two times with similar results. (B) Schematic of ligand droplets and sequential dual-lineage fibroblast seeding. (C) Fluorescence and brightfield microscopy images of GFP and mCherry droplets (left) and subsequent reporter expression (center) by dual-lineage fibroblasts after two days of culture on corresponding substrate. Scale bars, 2 mm. Green box (within GFP pattern) and red box (within mCherry pattern) are shown at higher magnification (right). Scale bars, 200 µm. (D) Brightfield image of dual-lineage fibroblasts cultured on droplet pattern for two days, demonstrating uniform coverage of engineered fibroblasts. Scale bar, 2 mm. (E) Fluorescence microscopy images of the same tissue shown in Fig. 7C without (left) and with (right) subtraction of GFP pattern. GFP was subtracted to improve visualization of α-actinin and VEGFR2 immunostaining by dual-lineage fibroblasts on microfluidic dual-ligand pattern. Scale bars, 1mm. (F) (left) Fluorescent microscopy images of dual-lineage fibroblasts on microfluidic dual-ligand pattern (immuno)stained for α-actinin (red), VEGFR2 (green), and nuclei (blue). (right) Profile plot of α-actinin and VEGFR2 immunosignal across x-axis. Green and red box indicate regions patterned with GFP and mCherry, respectively. Source data are provided as a Source Data file.

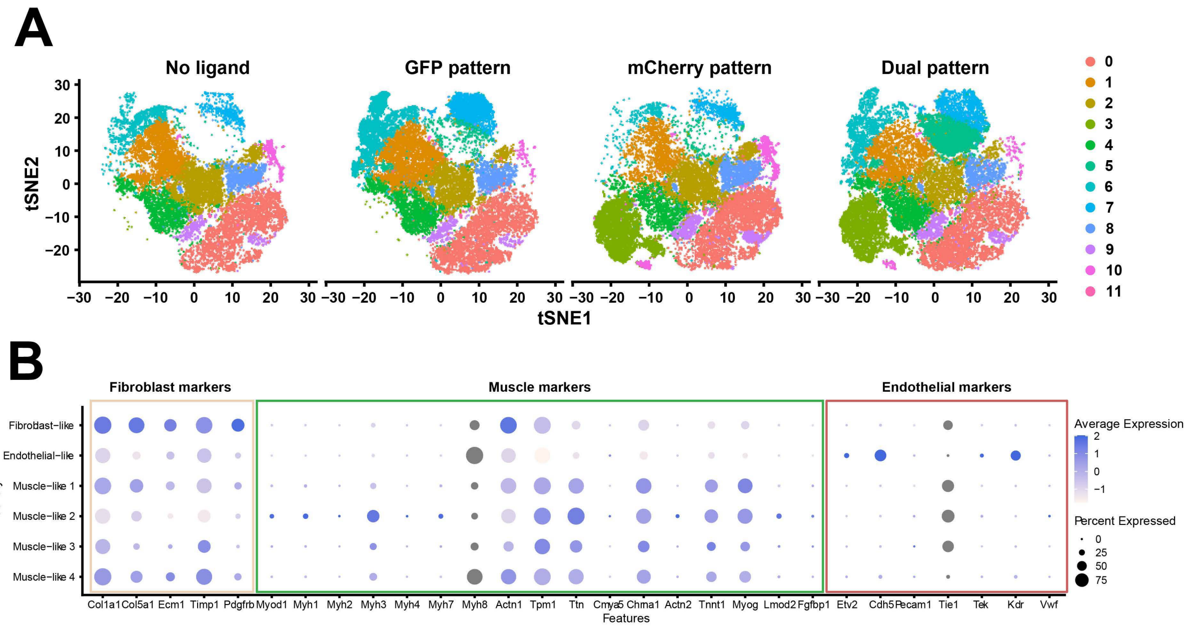
A
No ligand
GFP pattern
mCherry pattern
Dual pattern
tSNE2
tSNE1
B
Fibroblast markers
Muscle markers
Endothelial markers
Fibroblast−like
Endothelial−like
Muscle−like 1
Muscle−like 2
Muscle−like 3
Muscle−like 4
Identity
Col1a1 Col5a1 Ecm1 Timp1 Pdgfrb Myod1 Myh1 Myh2 Myh3 Myh4 Myh7 Myh8 Actn1 Tpm1 Ttn Cmya5 Chrna1 Actn2 Tnnt1 Myog Lmod2 Fgfbp1 Etv2 Cdh5 Pecam1 Tie1 Tek Kdr Vwf
Features
Average Expression
Percent Expressed

Figure S10. (A) T-Distributed Stochastic Neighbor Embedding (t-SNE) plot analysis showing twelve cell clusters based on gene expression profiles for anti-GFP and anti-mCherry synNotch dual-lineage fibroblasts with orthogonally inducible MyoD-miRFP and ETV2-BFP, respectively, cultured on all four indicated substrates micropatterned by microfluidics. (B) Selected marker gene analysis for fibroblast-like, endothelial-like, and the four muscle-like clusters. Color intensity represents average expression level and size of dot represents the percent of population expressing the marker. n=2 biological replicates per group sequenced independently.

Rank	Cluster 0	Cluster 1	Cluster 2	Cluster 3
1	**Cell Cycle**	Differentiation	Extracellular	Synapse
2	Chromosome	Calmodulin Binding	Heparin Binding	Cell Membrane
3	Mitosis	Cardiomyopathy	**Growth Factor**	PH
4	Microtubule Cytoskeleton	**Muscle Contraction**	Migration	Rho GTPase
5	Nucleus	Extracellular	Focal Adhesion	Ion Channel
6	Mitotic Assembly	**Sarcoplasmic Reticulum**	Innate Immunite	MAM
7	Microtubule	Actin Binding	Calcium binding	Presynaptic membrane
8	Nucleotide Binding	**Troponin**	Pathways	Cardiomyopathy
9	Mitotic Cell Cycle	Transmembrane	Collagen	SH3
10	Isopeptide Bond	HLH	kinase signaling	**Angiogenesis/ VEGF**
	(14) EGF		(11) EGF (13)MMP	
**Assigned Identity:**	Fibroblast-like Dividing	Muscle-like	Fibroblast-like	Endothelial-like

Cluster 4	Cluster 5	Cluster 6	Cluster 7	Cluster 8
Posysnaptic	Ribosome / Translation	Actin Binding	Ribosome	Cell Cycle
Differentiation	**Muscle Protein**	Cardiomyopathy	**Muscle Protein**	Chromsome
Membrane	**Troponin**	Differentiation	Cytoplasm	Mitosis
PH domain	Cytoplasm	**Sarcoplasmic Reticulum**	**Muscle Contraction**	Cell Cycle
Signaling Pathways	**Cardiac muscle contraction**	Fibronectin	**Muscle Contraction**	Cytoskeleton
Fibronectin	**Muscle Contraction**	PDZ	**Troponin**	Cell Cycle
SUSHI	EF-hand	PH domain	**Sarcoplasmic Reticulum**	ATP binding
**Extracellular Matrix**	Ribosomal	Calmodulin	Cardiomyopathy	Mitosis
**EGF**	Carboxylesterase	**Muscle Proteins**	Striated Muscle	Cyclin
Extracellular	Chemotaxis	**Troponin**	EF-hand	Isopeptide
Fibroblast	Muscle-like	Muscle-like	Muscle-like	Fibroblast-like Dividing

Cluster 9	Cluster 10	Cluster 11
Extrcellular Secreted	ribosome	Extracellular secreted
Cell Cycle	cytoplasm	Response to virus
**Extracellular matrix**	ribosome	**Extracellular matrix**
Chromosome	Isopeptide	Immune Signaling
Cytoskeleton	Splicesosome	Furin
Heparin-binding	Signal Recognition Particle	TSP1
**EGF-like**	EF-Hand1	GFP biding
Signal Pathway	Cell Cycle	Response to IFNb
**PDGF**	Oxidative Stress	Oxidative Stress
**EGF-like**	Antioxidant	TIR domain
Fibroblast-like	Fibroblast-like	Fibroblast-like

Table S1. DAVID pathway analysis and Assigned Identity of clusters. Pathways rank from 1-10, 1 being the pathway with most genes activated in its given pathway. Pathways in bold are associated with the cluster's assigned identity. For cluster 0 and 2, identity related pathways outside the top 10 were included with their rank in parenthesis. Each cluster was compared to the gene expression of all clusters combined together. List of genes were selected through filtering with $P < 0.01$ and $Log2FC > 0.5$. P-values determined via wilcoxon rank sum test.

RANK	Muscle 1 vs. Muscle 2 UP	Muscle 1 vs. Muscle 2 DOWN	Muscle 1 vs. Muscle 3 UP	Muscle 1 vs. Muscle 3 DOWN
1	Ribosome	Cardiomyopathy	Cell Projection	ribosome
2	Ribosomal	PH Domain	Cell Differentiation	cell cycle
3	Cytoplasm	Actin Binding	PH domain	ribosomal
4	Extracellular Secreted	Sarcoplasmic Reticulum	Calmodulin binding	Chromosome
5	Isopeptide Bond	PDZ	Hormone	Cytoskeleton
6	Collagen/PDGFR binding	RhoGTP	Rap1 Signaling	Isopeptide Bond
7	Signaling	Differentiation	Transcription	Mitosis
8	Iron	Muscle Protein	Cell Junction	GrowthFactor
9	Antioxidant	Fibronectin	PDZ	Microtubule
10	Signaling Pathway	SH3 Domain	SH3	EF-hand
	(14) EGF			

Muscle 1 vs. Muscle 4 UP	Muscle 1 vs. Muscle 4 DOWN	Muscle 2 vs. Muscle 3 UP	Muscle 2 vs. Muscle 3 DOWN
Developmental Protein	ribosome	PH domain	ribosome
Actin Binding	cell cycle	Cardiomyopathy	ribosome
Calmodulin Binding	ribosome	Actin Binding	cell cycle
Extracellular Matrix	extracellular secreted	PDZ	extracellular secreted
Cardiomyopathy	chromosome	SH3	isopeptide
Glycoprotein	actin binding	RhoGTP	extracellular matrix
PDZ	extracellular matrix	Cell Junction	mitosis
Sarcoplasmic Reticulum	Isopeptide bond	G8 Domain	mitosis
Fibronectin/IgG	EF-hand	SAM Domain	immune signaling
PH domain	Microtubule	Hormone	microtubule

Muscle 4 vs. Muscle 2 UP	Muscle 4 vs. Muscle 2 DOWN	Muscle 4 vs. Muscle 3 UP	Muscle 4 vs. Muscle 3 DOWN
Ribosome	actin binding	PH domain	sarcomere
Cell Cycle	differentiation	Extracellular matrix	actin binding
Ribosomal	sarcoplasmic reticulum	Extracellular secreted	sarcoplasmic reticulum
Extracellular Secreted	Fibronectin	CH dom	extracellular secreted
Differentiation	Ph domain	Rho GTPase	muscle contraction
PH domain	PDZ	Domain	muscle protein
Fibronectin	cardiomyopathy	Actin binding	signal
Actin Binding	muscle protein	Signaling Pathway	protein response
Chromosome	cell junction	Cell Cycle	cardiac contraction
Rho GTPase	SH3 domain	Membrane	peptide signaling
(13) Muscle Contraction			

Table S2. DAVID pathway analysis and comparison of UP and DOWN regulated pathways between all muscle-like clusters. Pathways rank from 1-10, 1 being the pathway with most genes UP or Down regulated between muscle-like clusters. Identity related pathways outside the top 10 were included with their rank in parenthesis. List of genes were selected through filtering with P < 0.01 and Log2FC > 0.5. P-values determined via wilcoxon rank sum test.

Company	Catalog	Target	Fluorophore	Dilution
Cell Signaling Technologies	3739S	Anti-Myc (EQKLISEEDL)	PE	FC: 1:50
Cell Signaling Technologies	15008S	Anti-FLAG (DYKDDDDK)	AlexaFluor 488	FC: 5uL/10^6 cells
Abcam	ab45688	Anti-Fibronectin		IF 1:500
BioLegend	136401	Anti-VEGFR2		IF: 1:100
BioLegend	136405	Anti-VEGFR2	APC	FC: 1:100
Sigma	A7811	Anti-α-Actinin (Sarcomeric)		IF: 1:200
ThermoFisher	14-1441-82	Anti-VE-cadherin		IF: 1:100
BioLegend	138005	Anti-VE-cadherin	AlexaFluor 647	FC: 1:100
ThermoFisher	A-11030	Anti-Mouse IgG	AlexaFluor 546	IF: 1:200
Abcam	ab150153	Anti-Rat IgG	AlexaFluor 488	IF: 1:200
ThermoFisher	A-2144	Anti-Rabbit IgG	AlexaFluor 647	IF: 1:1000
ThermoFisher	A-21235	Anti-Mouse IgG	AlexaFluor 647	IF 1:200
ThermoFisher	H10294	HSC NuclearMask Deep Red		IF: 1:250
Abcam	ab228551	Hoechst 33342		IF: 1:20,000
ThermoFisher	R37605	Hoechst 33342		IF: 2drops/mL

Table S3. Antibodies used in this study

pHR-TRE-mCherry
mCherry_F - CACAACACTTTTGTCTTATACTTAcgcgtGCCGCCACCatggtgagcaagggcgaggag
mCherry_R - atgttgcaggtgggagttgcggccgcTTACTTGTACAGCTCGTCCATGccg

pHR_EF1a_flagLaM4-Notch-Gal4VP64
Gal4VP64_F - gtgctgctgtcccgcaagcgccggcggatgaagctgct
Gal4VP64_R - atcaagcttgcatgcctgcaggtcgactctagag

pHR-TRE-miRFP703_PGK-Puro
PGK-Puro_F - TCATTGGTCTTAAAGTTTCTTTATGTTTTAAATGCACTGACCTCCCAC
PGK-Puro_R - ATTAATTCGAGCTCGGTACCTCTACCGGGTAGGGGAGG
miRFP_F - TCTTATACTTAcgcgtGCcgccaccatggtagcag
miRFP_R - gtgggagttgcggccgcttagctctcaagcgcggtgatcc

pHR-TRE-MyoD-P2A-miRFP703_PGK-Puro
PGK-Puro_F - TCATTGGTCTTAAAGTTTCTTTATGTTTTAAATGCACTGACCTCCCAC
PGK-Puro_R - ATTAATTCGAGCTCGGTACCTCTACCGGGTAGGGGAGG
MyoD-p2A_F - TCTTATACTTAcgcgtGCCGCCACCATGGAACTGCTCTCGC
MyoD-p2A_R - gctaccatAGGTCCAGGGTTCTCC
miRFP_F - TGGACCTatggtagcaggtcatgcctct
miRFP_R - gtgggagttgcggccgcttagctctcaagcgcggtgatcc

pHR-UAS-tagBFP_PGK-HygR
PGK-HygR_F - cgactctagagtcgacgggtaggggaggcgctt
PGK-HygR_R - ttccctcgaggtcgaccatagagcccaccgc
BFP_F - gacattcgttggatccGCCGCCaccatgagcgagctgattaagg
BFP_R - gtgggagttgcggccgcgcggggaggcgg

pHR-UAS-ETV2-p2A-tagBFP_PGK-HygR
PGK-HygR_F - cgactctagagtcgacgggtaggggaggcgctt
PGK-HygR_R - ttccctcgaggtcgaccatagagcccaccgc
ETV2_F - ctctcgacattcgttggatccGCCACCatggacctgtggaact
ETV2_R - CTCCGCTTCCttggccttctgcacctgg
p2A-BFP_F - agaaggccaaGGAAGCGGAGCTACTAACTTCAG
p2A-BFP_R - tgcaggtgggagttgcggccgcgcggggaggcgg

Mouse ETV2
MDLWNWDEASLQEVPPGDKLTGLGAEFGFYFPEVALQEDTPITPMNVEGCWKGFPELDWNPALP
HEDVPFQAEPVAHPLPWSRDWTDLGCNTSDPWSCASQTPGPAPPGTSPSPFVGFEGATGQNPA
TSAGGVPSWSHPPAAWSTTSWDCSVGPSGATYWDNGLGGEAHEDYKMSWGGSAGSDYTTTW
NTGLQDCSIPFEGHQSPAFTTPSKSNKQSDRATLTRYSKTNHRGPIQLWQFLLELLHDGARSSCIR
WTGNSREFQLCDPKEVARLWGERKRKPGMNYEKLSRGLRYYYRRDIVLKSGGRKYTYRFGGRVP
VLAYQDDMGHLPGAEGQ*

Oligonucleotides used in this study.

```
// start code
// Custom shuffle function
function shuffleArray(array) {
    for (i = array.length - 1; i > 0; i--) {
        j = floor(random() * (i + 1));
        temp = array[i];
        array[i] = array[j];
        array[j] = temp;
    }
    return array;
}

// Get the current image
image = getImageID();
selectImage(image);
getDimensions(width, height, channels, slices, frames);

// Get the pixel values
array = newArray(width * height);
for (y = 0; y < height; y++) {
    for (x = 0; x < width; x++) {
        index = x + y * width;
        array[index] = getPixel(x, y);
    }
}

// Shuffle the array
array = shuffleArray(array);

// Create a new image to hold the scrambled pixels
newImage("Scrambled Image", "8-bit black", width, height, 1);
selectWindow("Scrambled Image");

// Set the scrambled pixels
for (y = 0; y < height; y++) {
    for (x = 0; x < width; x++) {
        index = x + y * width;
        setPixel(x, y, array[index]);
    }
}

// Show the scrambled image
updateDisplay();
// end code
```

Image pixel shuffler code for ImageJ
