# Supplementary material for: Engineering programmable material-to-cell pathways via synthetic notch receptors to spatially control differentiation in multicellular constructs
Source: Nat Commun. 2024 Jul 13;15:5891. doi: 10.1038/s41467-024-50126-1 (PMC11246427; doi:10.1038/s41467-024-50126-1)
Supplement: Supplementary file 2 — Reporting Summary [file 41467_2024_50126_MOESM2_ESM.pdf]

nature portfolio

Corresponding author(s): *Double-blind peer review submissions: write DBPR and your manuscript number here instead of author names.*

Last updated by author(s): *YYYY-MM-DD*

Reporting Summary

Nature Portfolio wishes to improve the reproducibility of the work that we publish. This form provides structure for consistency and transparency in reporting. For further information on Nature Portfolio policies, see our Editorial Policies and the Editorial Policy Checklist.

Statistics

For all statistical analyses, confirm that the following items are present in the figure legend, table legend, main text, or Methods section.

n/a	Confirmed	
☐	☒	The exact sample size (n) for each experimental group/condition, given as a discrete number and unit of measurement
☐	☒	A statement on whether measurements were taken from distinct samples or whether the same sample was measured repeatedly
☐	☒	The statistical test(s) used AND whether they are one- or two-sided<br>*Only common tests should be described solely by name; describe more complex techniques in the Methods section.*
☒	☐	A description of all covariates tested
☒	☐	A description of any assumptions or corrections, such as tests of normality and adjustment for multiple comparisons
☒	☐	A full description of the statistical parameters including central tendency (e.g. means) or other basic estimates (e.g. regression coefficient) AND variation (e.g. standard deviation) or associated estimates of uncertainty (e.g. confidence intervals)
☒	☐	For null hypothesis testing, the test statistic (e.g. F, t, r) with confidence intervals, effect sizes, degrees of freedom and P value noted<br>*Give P values as exact values whenever suitable.*
☒	☐	For Bayesian analysis, information on the choice of priors and Markov chain Monte Carlo settings
☒	☐	For hierarchical and complex designs, identification of the appropriate level for tests and full reporting of outcomes
☐	☒	Estimates of effect sizes (e.g. Cohen's d, Pearson's r), indicating how they were calculated

Our web collection on statistics for biologists contains articles on many of the points above.

Software and code

Policy information about availability of computer code

Data collection	Built-in BZ-X Keyence software was used to image as well as tiling images taken by Keyence. Zeiss LSM880 and its software were also used for imaging. Illumina NovaSeq6000 and its software were used for bulk RNA seq (performed by Novogene). Illumina NovaSeqX sequencer was used for single-nuclei sequencing (performed by UCSF CAT).
Data analysis	ImageJ v1.54d used for image analysis and quantification, plugins: JACoP, OrientationJ, and published cell-segmentation based Macro (Nat. Mater. 21, 1191–1199 (2022)). Bulk RNA-seq was analyzed using Trimmomatic v0.39, STAR v2.7.10b, DESEq2 v1.38.3, and clusterProfiler v4.6.2. Single-nuclei sequencing was analyzed using Qubit, BioAnalyzer, 10X Genomics' Cell Ranger, and Seurat v5.0.2.

For manuscripts utilizing custom algorithms or software that are central to the research but not yet described in published literature, software must be made available to editors and reviewers. We strongly encourage code deposition in a community repository (e.g. GitHub). See the Nature Portfolio guidelines for submitting code & software for further information.

Data

Policy information about availability of data

All manuscripts must include a data availability statement. This statement should provide the following information, where applicable:

- Accession codes, unique identifiers, or web links for publicly available datasets
- A description of any restrictions on data availability
- For clinical datasets or third party data, please ensure that the statement adheres to our policy

The experimental raw data used to compile the graphs shown in the figures are available in Figshare with identifier https://doi.org/10.6084/m9.figshare.25648026.

The results of the sequencing experiments are being uploaded to the appropriate repositories in NIH GEO; links will be available by the next submission.
All the rest of the data supporting the findings of this study are available within the paper and its Supplementary Information.

Human research participants

Policy information about studies involving human research participants and Sex and Gender in Research.

Reporting on sex and gender	*Use the terms sex (biological attribute) and gender (shaped by social and cultural circumstances) carefully in order to avoid confusing both terms. Indicate if findings apply to only one sex or gender; describe whether sex and gender were considered in study design whether sex and/or gender was determined based on self-reporting or assigned and methods used. Provide in the source data disaggregated sex and gender data where this information has been collected, and consent has been obtained for sharing of individual-level data; provide overall numbers in this Reporting Summary. Please state if this information has not been collected. Report sex- and gender-based analyses where performed, justify reasons for lack of sex- and gender-based analysis.*
Population characteristics	*Describe the covariate-relevant population characteristics of the human research participants (e.g. age, genotypic information, past and current diagnosis and treatment categories). If you filled out the behavioural & social sciences study design questions and have nothing to add here, write "See above."*
Recruitment	*Describe how participants were recruited. Outline any potential self-selection bias or other biases that may be present and how these are likely to impact results.*
Ethics oversight	*Identify the organization(s) that approved the study protocol.*

Note that full information on the approval of the study protocol must also be provided in the manuscript.

Field-specific reporting

Please select the one below that is the best fit for your research. If you are not sure, read the appropriate sections before making your selection.

[x] Life sciences [ ] Behavioural & social sciences [ ] Ecological, evolutionary & environmental sciences

For a reference copy of the document with all sections, see nature.com/documents/nr-reporting-summary-flat.pdf

Life sciences study design

All studies must disclose on these points even when the disclosure is negative.

Sample size	Sample sizes > 3 were chosen to give statistical significance.
Data exclusions	No data was excluded in this study,
Replication	*Describe the measures taken to verify the reproducibility of the experimental findings. If all attempts at replication were successful, confirm this OR if there are any findings that were not replicated or cannot be reproduced, note this and describe why.*
Randomization	*Describe how samples/organisms/participants were allocated into experimental groups. If allocation was not random, describe how covariates were controlled OR if this is not relevant to your study, explain why.*
Blinding	*Describe whether the investigators were blinded to group allocation during data collection and/or analysis. If blinding was not possible, describe why OR explain why blinding was not relevant to your study.*

Reporting for specific materials, systems and methods

We require information from authors about some types of materials, experimental systems and methods used in many studies. Here, indicate whether each material, system or method listed is relevant to your study. If you are not sure if a list item applies to your research, read the appropriate section before selecting a response.

Materials & experimental systems

n/a	Involved in the study	
☐	☒	Antibodies
☐	☒	Eukaryotic cell lines
☒	☐	Palaeontology and archaeology
☒	☐	Animals and other organisms
☒	☐	Clinical data
☒	☐	Dual use research of concern

Methods

n/a	Involved in the study	
☒	☐	ChIP-seq
☐	☒	Flow cytometry
☒	☐	MRI-based neuroimaging

Antibodies

Antibodies used

Cell Signaling Technologies 3739S Anti-Myc-546 (EQKLISEEDL)
Cell Signaling Technologies 15008S Anti-FLAG-488 (DYKDDDDK)
Abcam ab45688 Anti-Fibronectin
BioLegend 136401 Anti-VEGFR2
BioLegend 136405 Anti-VEGFR2-APC
Sigma A7811 Anti-α-Actinin (Sarcomeric)
ThermoFisher A-11030 Anti-Mouse IgG AlexaFluor 546
Abcam ab150153 Anti-Rat IgG AlexaFluor 488
ThermoFisher A-2144 Anti-Rabbit IgG AlexaFluor 647
ThermoFisher A-21235 Anti-Mouse IgG AlexaFluor 647
ThermoFisher H10294 HSC NuclearMask Deep Red

Validation

All flow cytometry and cell sorting antibodies were validated with stained vs. unstained controls
All Immunofluorescence staining were validated with samples stained only with secondary antibodies (no primary) to confirm specificity.

Eukaryotic cell lines

Policy information about cell lines and Sex and Gender in Research

Cell line source(s)

L929 mouse fibroblast cells (ATCC# CCL-1), HEK293 cells (Takara 632180), C3H/10T1/2 Clone 8 (ATCC# CCL-226), NIH/3T3 (ATCC# CRL-1658), C2C12 (ATCC# CRL-1772), and bEnd.3 (ATCC# CRL-2299)

Authentication

Cell lines were not authenticated.

Mycoplasma contamination

All cell lines were tested for mycoplasma contamination and were negative.

Commonly misidentified lines (See ICLAC register)

Name any commonly misidentified cell lines used in the study and provide a rationale for their use.

Flow Cytometry

Plots

Confirm that:

☒ The axis labels state the marker and fluorochrome used (e.g. CD4-FITC).

☒ The axis scales are clearly visible. Include numbers along axes only for bottom left plot of group (a 'group' is an analysis of identical markers).

☒ All plots are contour plots with outliers or pseudocolor plots.

☒ A numerical value for number of cells or percentage (with statistics) is provided.

Methodology

Sample preparation

Cells were detached using TrypLE (ThermoFisher) and washed once prior to incubation with fluorescently-tagged antibodies in PBS+5%FBS for 30 minutes-1 hour at 4°C. Following, cells were washed twice with PBS+5%FBS and filtered through 35µm cell strainer prior to analysis with ARIA II.

Instrument

Aria II (Beckton-Dickinson)

Software

FlowJo was used to analyze data

Cell population abundance

Percentage of positively-expressing or positively-stained cells were calculated based on the percent compared to a gated single cell whole population

Gating strategy	Forward Scatter-Area and Side Scatter-Area were used to separate expected cell clusters from debris and larger cell aggregates. Singlets were selected using Forward Scatter-Height and Forward Scatter-Width. Percent positively expressing cells were determined using the same cells lines in their unactivated state, or unstained controls of activated cells.

☒ Tick this box to confirm that a figure exemplifying the gating strategy is provided in the Supplementary Information.
